# Combinatorial Pharmacophore-Based 3D-QSAR Analysis and Virtual Screening of FGFR1 Inhibitors

**DOI:** 10.3390/ijms160613407

**Published:** 2015-06-11

**Authors:** Nannan Zhou, Yuan Xu, Xian Liu, Yulan Wang, Jianlong Peng, Xiaomin Luo, Mingyue Zheng, Kaixian Chen, Hualiang Jiang

**Affiliations:** 1 State Key Laboratory of Bioreactor Engineering and Shanghai Key Laboratory of Chemical Bilolgy, School of Pharmacy, East China University of Science and Technology, Shanghai 200237, China; E-Mail: nannanzhou0912@163.com; 2 State Key Laboratory of Drug Research, Shanghai Institute of Materia Medica, Chinese Academy of Sciences, 555 Zuchongzhi Road, Shanghai 201203, China; E-Mails: xu_yuan88@163.com (Y.X.); xliu@ms.iswc.ac.cn (XianL.); ylwang@simm.ac.cn (Y.W.); pjlxsda@163.com (J.P.); xmluo@simm.ac.cn (XiaoL.); kxchen@simm.ac.cn (K.C.)

**Keywords:** pharmacophore, combinatorial 3D-QSAR, virtual screening, FGFR1 inhibitors

## Abstract

The fibroblast growth factor/fibroblast growth factor receptor (FGF/FGFR) signaling pathway plays crucial roles in cell proliferation, angiogenesis, migration, and survival. Aberration in FGFRs correlates with several malignancies and disorders. FGFRs have proved to be attractive targets for therapeutic intervention in cancer, and it is of high interest to find FGFR inhibitors with novel scaffolds. In this study, a combinatorial three-dimensional quantitative structure-activity relationship (3D-QSAR) model was developed based on previously reported FGFR1 inhibitors with diverse structural skeletons. This model was evaluated for its prediction performance on a diverse test set containing 232 FGFR inhibitors, and it yielded a SD value of 0.75 pIC_50_ units from measured inhibition affinities and a Pearson’s correlation coefficient *R*^2^ of 0.53. This result suggests that the combinatorial 3D-QSAR model could be used to search for new FGFR1 hit structures and predict their potential activity. To further evaluate the performance of the model, a decoy set validation was used to measure the efficiency of the model by calculating *EF* (enrichment factor). Based on the combinatorial pharmacophore model, a virtual screening against SPECS database was performed. Nineteen novel active compounds were successfully identified, which provide new chemical starting points for further structural optimization of FGFR1 inhibitors.

## 1. Introduction

Fibroblast growth-factor receptors (FGFRs), a sub-family of the receptor tyrosine kinase (RTK) superfamily, play critical roles in many physiologic process such as embryogensis, cell proliferation and migration, and angiogenesis by binding with fibroblast growth factors (FGF) and heparan sulfate proteoglycans (HPSG) [1]. FGFRs (FGFR1-4) are single-pass, transmembrane, tyrosine kinase receptors. The cytoplaxmic portion contains a conserved tyrosine kinase domain and a COOH tail, while the extracellular domain contains an acidic box containing eight consecutive acidic residues and three immunoglobulin-like (Ig) fragments that Ig-I, together with the acidic box, involves in receptor auto-inhibition, Ig-II and Ig-III form FGF and HPSG binding domains. When binding to FGF and HPSG, FGFRs perform dimerization and then cause autophosphorylation and substrate phosphorylation on tyrosine. Subsequently, those phosphorylations trigger the cascade reactions of several signal transduction pathways, such as phospholipase-γ (PLC-γ) pathway and mitogen-activated protein kinase (MAPK) pathway, and finally regulate a variety of physiological processes [2,3]. It has been shown that aberrant FGF signaling is involved in the development of tumors such as bladder cancer, lung cancer, gastric cancer, endometrial cancer and so on [4]. Accordingly, FGFR is an important target for cancer treatment. In recent years, there have been a variety of compounds reported as FGFR inhibitors [5,6,7,8,9,10], and many NCEs (new chemical entities) have been in clinical development such as TKI258 [11], E-3810 [12], BGJ398 [13] and so on. The most widely designed inhibitors are small competitive inhibitors targeting the ATP-binding site of the cytoplaxmic tyrosine kinase domain through hydrogen bonds or polar interactions. For any further information, one may refer to the following excellent reviews [1,4,14,15,16].

Although a large group of promising compounds has been synthesized, the inhibition activities of new designed molecules against FGFR could not be conformed until experimentally validated, which are time-consuming and expensive. Therefore, it is necessary to develop consistent *in silico* tools for activity prediction. Pharmacophore and QSAR model have become important tools in computer-aided drug design such as virtual screening and lead optimization. In this study, we focus on a new combinatorial 3D-QSAR model for activity prediction.

A pharmacophore model can be built either in a (target-) structure-based manner or a ligand-based manner. Structure-based pharmacophore is based on the apo protein structure or protein-ligand complex, which needs to analyze the complementary chemical features of activities site and their spatial relationships, and then to build pharmacophore assembly with selected features. The limitation of this kind of model is that too many chemical features can be identified to apply for practical applications. Additionally, it cannot reflect the quantitative structure-activity relationship (QSAR) as it just considers a single target or a single target-ligand complex [17]. Compared with structure-based model, ligand-based pharmacophore is more frequently used, which extracts common chemical features from aligned compound structures interacting with the same target, based on the hypothesis that compounds interacting with the same protein target may share similar chemical structure and physicochemical properties [18,19]. The pivotal issues of the ligand-based model are the modeling of ligand flexibility, the alignment methods of molecules and the selection of training set. Different pharmacophore models could be derived from different training sets because it is easily affected by the type of the ligand, the site of the dataset and chemical diversity [17].

QSAR model, which quantifies the correlation between structures of a series of compounds and biological activities, is based on the hypothesis that compounds with similar structures or physiochemical properties have similar activities [20]. The development of a QSAR model involves a series of consecutive steps, including: (1) Collect ligands with known activity with the same target; (2) Extract descriptors representing the molecule; (3) Select best descriptors from a larger set of descriptors; (4) Map the molecular descriptors into the biological activity; and (5) Internal and external validation of the QSAR model [21]. Compared with classical QSAR method using fragment-based descriptors such as electronic, hydrophobic and steric features, 3D-QSAR model is based on 3D descriptors such as various geometric, physical characteristics and quantum chemical descriptors, which are useful in describing the ligand-receptor interactions [22]. Statistical tools such as multivariable linear regression analysis (MLR), principal component analysis (PCA) and partial least square analysis (PLS) can be used for linear QSAR modeling, while there are also many non-linear models established using neural network, Bayesian neural network and others machine learning techniques. To validate the QSAR model, internal cross validation is used and to calculate the cross validated *R*^2^ or *Q*^2^. Besides that, external validation is also necessary, which is performed by predicting the activity of external test sets not used for model building [20]. These 3D-QSAR models have done well when the molecules used for model developing are based on congeneric series of molecules [23,24,25].

Since QSAR model is more suitable for congeneric series of molecules, developing a single model for various chemical structures like inhibitors of FGFR with diverse core skeletons is challenging. In an attempt to design novel chemical entities with FGFR1 inhibition activities, in this study, we performed pharmacophore modeling and 3D-QSAR analyses for different chemical classes of FGFR1 inhibitors, and developed a combinatorial pharmacophore-based 3D QSAR model. For each class of inhibitors, 4-point, 5-point and 6-point pharmacophore hypotheses were generated, respectively, using the active compounds in a training set. Then, alignment conformations of all compounds were generated for subsequent 3D-QSAR modeling [26]. The model was validated by an external test set and a decoys set. Furthermore, virtual screening protocol based on the resulted combinatorial pharmacophore model was performed against a commercial chemical database. Finally, several compounds were short-listed as potential FGFR1 inhibitors by experimental evaluation with biological assay of a fraction of top ranked compounds.

## 2. Results and Discussion

**Figure 1:**
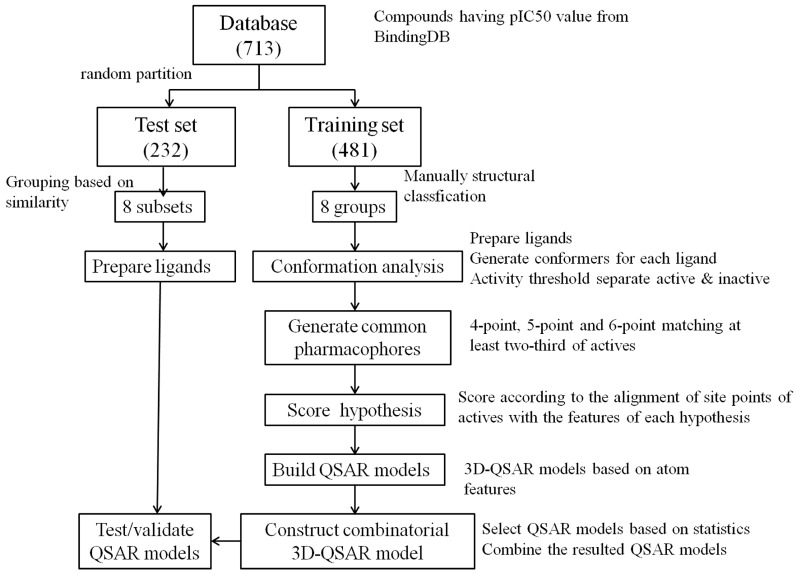
The flowchart to develop the combinatorial pharmacophore-based three-dimensional quantitative structure-activity relationship (3D-QSAR) model.

Figure 1 shows the flowchart of developing the combinatorial 3D-QSAR model. In each group of inhibitors, hundreds of pharmacophore hypotheses that would be subsequently used for QSAR modeling were generated. The final 3D-QSAR model of each group was selected based on a series of statistic parameters, including *R*^2^, *F*, *p* and stability. *R*^2^ is the mean value of square of the correlation coefficient of regression. *F* is the ratio of model variance to the observed activity variance and a larger *F* indicates a more statistically significant regression. *p* is significance level of variance ratio and smaller values represent a greater degree of confidence. Stability value reflects the stability of the model predictions with changes in the training set composition. Therefore, an ideal QSAR model should have large *R*^2^, small standard deviation (SD), large *F*, small *p* and large stability. Table 1 lists statistic parameters of the combinatorial QSAR model. The *R*^2^ and stability value of all groups are above 0.8 and 0.5, respectively, suggesting that all the components of the combinatorial model are stable and present good correlation between the experimental and predicted values.

**Table 1:** Statistic parameters of the combinatorial QSAR model for the training set.

Group	Number of Compounds	*R*^2^	SD	*F*	*p*	Stability
1 (ARRR)	83	0.81	0.41	114.50	7.35 × 10^−29^	0.51
2 (ADRRR)	29	0.89	0.32	75.30	3.10 × 10^−13^	0.61
3 (AAAARR)	62	0.86	0.32	121.70	5.14 × 10^−25^	0.74
4 (DDRRR)	99	0.81	0.38	134.70	2.82 × 10^−34^	0.53
5 (ADHRR)	18	0.99	0.09	362.60	1.67 × 10^−13^	0.53
6 (ADDH)	25	0.96	0.14	170.90	6.58 × 10^−15^	0.89
7 (ADHR)	20	0.98	0.15	237.80	1.78 × 10^−13^	0.56
8 (DRRRR)	39	0.98	0.21	649.80	9.81 × 10^−31^	0.85

**Figure 2:**
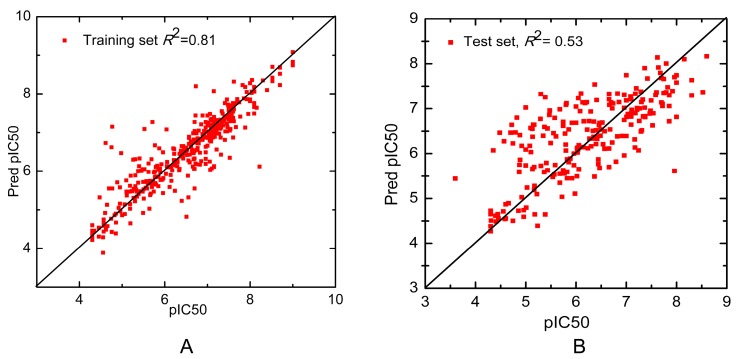
Scatter plot of the observed *versus* predicted activity generated by the combinatorial 3D-QSAR model of (**A**) the training set and (**B**) the test set.

It should be noted that the predictive ability of the whole dataset rather than each subset is of more interests. Figure 2 shows the scatter plots of the whole training set and test set. The *R*^2^ of training set and test set are 0.81 and 0.53, respectively, showing that our model could well reflect the linear relationship of the experimental activities and the predicted ones. Additionally, each individual QSAR model was used to predict the entire test set of 232 compounds and the prediction performance of these individual QSAR models were compared to that of the combinatorial QSAR model. As listed in Table 2, the *R*^2^ and the SD of the combinatorial QSAR model is higher and lower than any other single QSAR model, respectively, suggesting that the combinatorial QSAR model has advantage over any single QSAR models when predicting the whole test set. Besides, all the *R*^2^ of each individual model on classified subsets are larger than that on the whole test set. The potential reason is that QSAR models are more effective for congeneric series of molecules and the predictive ability decreases as the structural or physicochemical properties of new molecules varies from that in the training set [20]. This phenomenon suggests that the combinatorial QSAR model is more appropriate for activity prediction of structurally diversified compounds. Based on their similarity to each group of training data, our method predicts a variety of molecules using different QSAR models, which successfully avoid the above disadvantages.

**Table 2:** Prediction performance of single QSAR model and combinatorial QSAR model on test set.

Group	Test Set Grouped Based on Similarity			Total Test Set (232)		
	Matched Hits	*R*^2^	SD	Matched Hits	*R*^2^	SD
1 (ARRR)	51	0.53	0.64	229	0.00 *	1.12
2 (ADRRR)	16	0.37	0.71	231	0.00 *	1.16
3 (AAAARR)	29	0.41	0.68	227	0.01	1.09
4 (DDRRR)	43	0.17	0.87	227	0.00 *	1.12
5 (ADHRR)	9	0.66	0.79	232	0.00 *	1.09
6 (ADDH)	16	0.54	0.68	184	0.00 *	1.00
7 (ADHR)	23	0.37	0.90	216	0.03	1.10
8 (DRRRR)	42	0.87	0.33	229	0.12	1.01
Combinatorial QSAR Model	–	–	–	229	0.53	0.75

* indicates that the *R*^2^ is less than 0.01.

**Figure 3:**
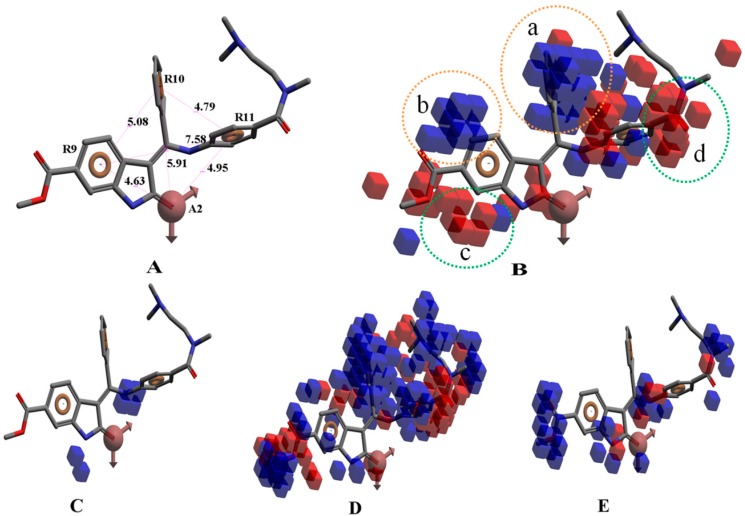
(**A**) The hypothesis (ARRR) that yielded the 3D-QSAR model of group 1; (**B**) Visualization of the 3D-QSAR model of group 1; Decomposed effects from (**C**) H-bond donor; (**D**) Hydrophobic/non-polar and (**E**) Electron-withdrawing of reference ligand (BindingDB50279269). The interaction is represented by clouded cubes where the blue ones indicate a favorable effect and the red ones indicate unfavorable effects. The colored circles indicate favorable and unfavorable interaction domains of the 3D-QSAR model.

Take group 1 as an example, the predictive 3D-QSAR model and the associated four-point hypothesis are shown in Figure 3, which contains one hydrogen bond acceptor and three aromatic ring features. The core structure of this group is oxindole, which is one of the first discovered structural category of FGFR inhibitors [15]. The ketonic oxygen of the reference ligand (BindingDB50279269) was mapped to the hydrogen bind acceptor, while three phenyl groups mapped to aromatic ring features.

**Figure 4:**
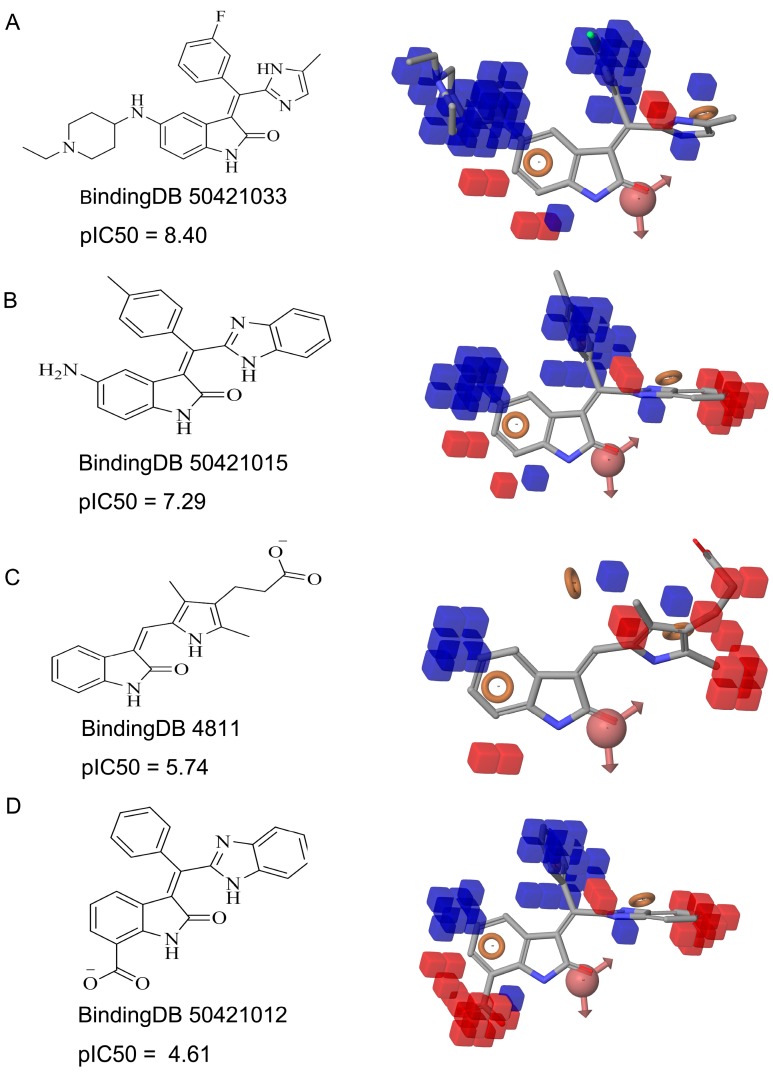
The QSAR model visualized in the context of the most active (**A**); moderately active (**B**,**C**); and the least active (**D**) molecules in training set.

Figure 3B shows the 3D-QSAR model containing two favorable (domain **a**, domain **b**) and two unfavorable (domain **c**, domain **d**) domains. The favorable and unfavorable interactions are combined effects of different interaction types such as H-bond donor, hydrophobic/non-polar and so on. In order to understand whether a specific feature is favored or disfavored to activity, we decomposed the interaction shown in Figure 3B into the effects from (Figure 3C) H-bond donor, (Figure 3D) hydrophobic/non-polar and (Figure 3E) electron-withdrawing. Given the reference ligand BindingDB50279269, we may find that two H-bond donors, N-1 of the oxindole and the N on the side chain of C-3 oxindole, are just located close to the H-bond donor favorable regions, which may explain the good activity of the compound. Moreover, a survey of existing crystal structures (PDB: 1AGW and PDB 1FGI) [27] revealed that the hydrogen bond interactions are indeed present at these regions. The favorable domain **a** and unfavorable domain **d** shown in Figure 3B correspond to hydrophobic interactions, by comparing the contour maps of Figure 3B,D. The electron-withdrawing effects shown in Figure 3E suggested that the methyl acetate group on the C-6 of oxindole structure is beneficial to activity. Figure 4 illustrates favorable and unfavorable interactions of several compounds mapped to the 3D-QSAR model. Compound BindingDB50421033 (Figure 4A) well occupies two favorable domains and has fewer interactions at unfavorable domains, which shows the highest activity among the training set. Compound BindingDB504210112 (Figure 4D), which, taking up two unfavorable domains (unfavorable domain **c** and **d**), is the least active molecule. Intuitively, activity will decrease when molecules take up the unfavorable domain **c**, as reflected by comparing BindingDB50421015 (Figure 4B) and BindingDB50421012 (Figure 4D). The absence of favorable domain **a** leads to the activity decrease in comparison of BindingDB50421015 (Figure 4B) and BindingDB4811 (Figure 4C). Sun *et al.* [28] reported that a substitution of electron-withdrawing groups on the phenyl ring of the oxindole can improve the inhibitory activity, which is consistent with the conclusion that the domain **b** has a positive contribution for maintaining the activity.

**Figure 5:**
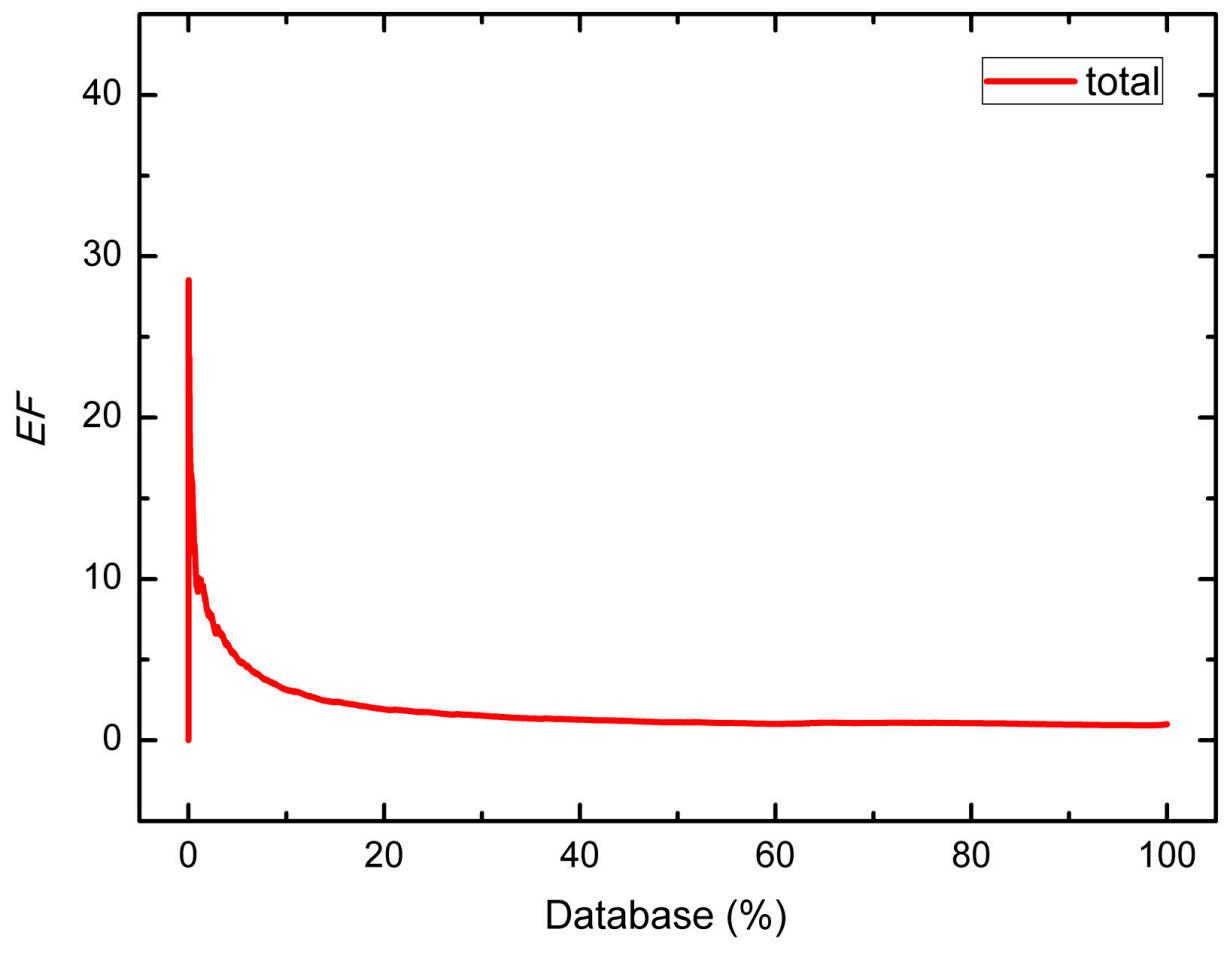
*EF* curve of the whole decoys database.

A decoy set of 7897 compounds including 232 inhibitors was used to further evaluate the ability of this combinatorial model to identify actives from a relatively large dataset. As shown in Table 3, the maximum *EF* values of all groups appear at 1%–2%, meaning that when we screen the database, true positive compounds can be efficiently recognized among the top ranked compounds. Figure 5 shows the *EF* curve of the combinatorial QSAR model against the whole decoy dataset. The curve shows a peak when the percent of database screened is less than 5%, illustrating that our model is suitable for screening potential actives from a large database.

**Table 3:** Enrichment factor (*EF*) values of each decoy dataset.

Group	Number of Hits/Size of Dataset			*EF*			
	Decoy Set	Decoys	Inhibitors	1%	2%	5%	10%
1	2063/2804	2012/2750	51/54	11.56	7.89	5.50	3.34
2	836/915	820/899	16/16	26.13	18.44	11.20	6.84
3	1048/1121	1019/1092	29/29	14.46	10.33	8.34	6.54
4	629/716	586/673	43/43	14.63	14.63	12.74	8.59
5	737/773	728/764	9/9	0.00	10.92	6.64	3.32
6	93/109	77/93	16/16	5.81	5.81	4.65	3.49
7	542/593	519/570	23/23	23.57	17.14	9.60	4.80
8	585/866	543/824	42/42	9.29	4.64	2.40	2.60
Total	6533/7897	6304/7665	229/232	10.09	7.84	5.06	3.15

**Figure 6:**
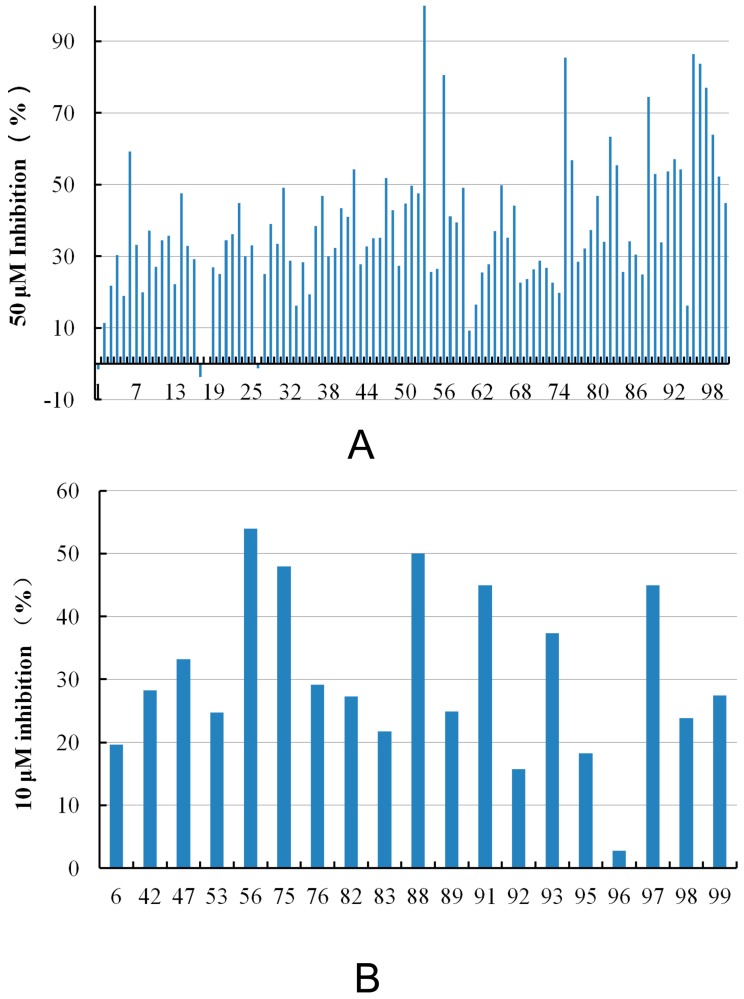
Inhibition ratio of FGFR1 at (**A**) 50 μM and (**B**) 10 μM compound concentrations.

One application of our model is to perform virtual screening to find hits with desired activity from a large chemical database. After screening the SPECS database, 100 compounds were purchased for biological assay, and those with potential PAINS (Pan Assay Interference Compounds) structures [29] were excluded. Figure 6 illustrates the results of experimental assays. The selected compounds were firstly screened at 50 μM concentrations *in vitro* FGFR1 kinase assays, and 19 of them displayed ≥50% inhibition of FGFR1 at 50 μM compound concentration. Then, the 19 compounds were further tested at 10 μM compound concentration, and five compounds display ≥40% FGFR1 inhibition, *i.e.*, compound **56**, **75**, **88**, **91**, **97**. Table 4 shows the structure and the inhibition ratios of these active compounds. To investigate the structural novelty of the hit compounds, we further compared the similarity between the hits and the all the reported active FGFR1 compounds from BindingDB. For each hit compound, the structure of the most similar known FGFR1 inhibitor in BindingDB (and the corresponding FCFP4 similarity) is list in Table 4. The low similarity values highlighted that the hits identified in this study are of excellent structural novelty. In the end, the five compounds display ≥40% FGFR1 inhibition were further evaluated to determine their IC_50_ values against FGFR1 kinase. Among them, compounds **88**, **91** and **97** rapidly precipitated at higher concentrations due to poor solubility. Compounds **56** and **75** showed IC_50_ values of 7.9 and 55.5 μM, respectively.

**Table 4:** FGFR1 enzyme inhibition rate of hit compounds, and the corresponding most similar compounds from BindingDB.

Compound ID	Hit Compound Structure	Inhibition (%) ^a^		Similar Structure in BindingDB	Similarity ^b^
		50 μM	10 μM		
**6**	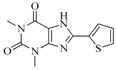	59.20	19.65	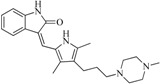	0.25
				BindingDB4812	
**42**	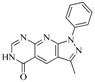	54.20	28.25	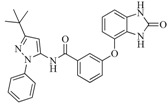	0.27
				BindingDB50307880	
**47**	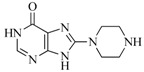	51.80	33.20	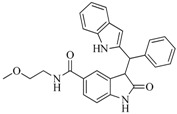	0.23
				BindingDB50421018	
**53**	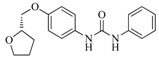	104.30	24.75	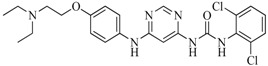	0.37
				BindingDB50234144	
**56**	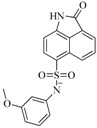	80.60	54.00	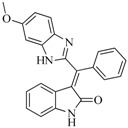	0.32
				BindingDB50420994	
**75**	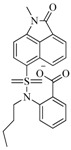	85.40	48.00	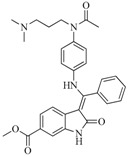	0.26
				BindingDB50279238	
**76**	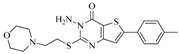	56.80	29.15	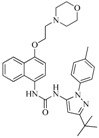	0.29
				BindingDB13533	
**82**	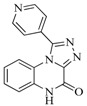	63.40	27.30	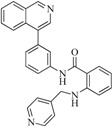	0.35
				BindingDB50121980	
**83**	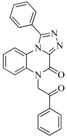	55.40	21.80	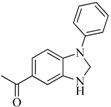	0.32
				BindingDB3855	
**88**	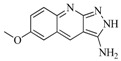	74.50	50.00	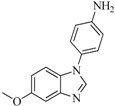	0.35
				BindingDB3933	
**89**	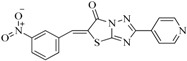	53.00	24.95	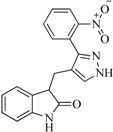	0.30
				BindingDB50431812	
**91**	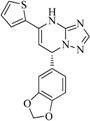	53.70	45.00	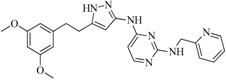	0.30
				BindingDB50420968	
**92**	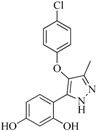	57.10	15.75	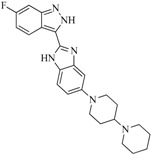	0.25
				BindingDB50185172	
**93**	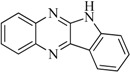	54.30	37.35	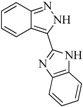	0.38
				BindingDB50185180	
**95**	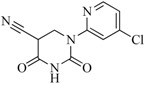	86.40	18.30	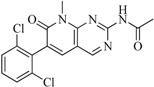	0.28
				BindingDB3051	
**96**	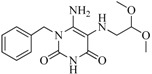	83.70	2.80	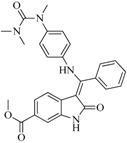	0.28
				BindingDB50279045	
**97**	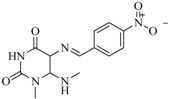	77.10	45.00	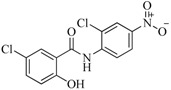	0.28
				BindingDB11242	
**98**	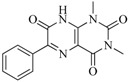	64.00	23.85	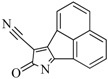	0.28
				BindingDB50345445	
**99**	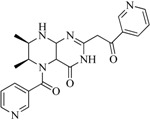	52.30	27.45	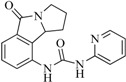	0.27
				BindingDB6619	

^a^ Inhibition of FGFR1 activity at 50 and 10 μM concentrations of listed compounds. The results present an average value of two independent experiments performed in duplicate; ^b^ Similarities between hits and the corresponding most similarity compounds from BindingDB.

## 3. Experimental Section

### 3.1. Dataset

**Figure 7:**
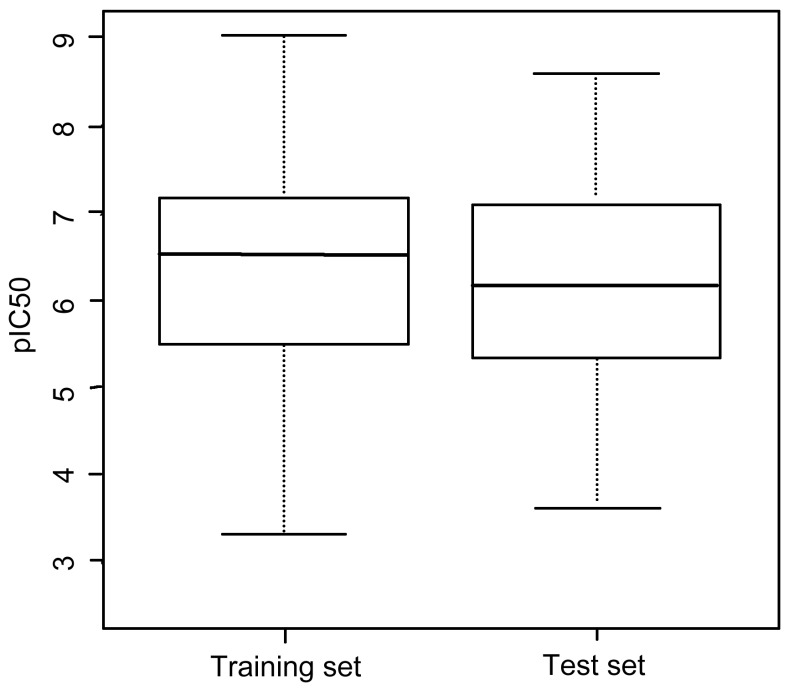
Biological activity range of training set and test set.

A reasonable dataset is of great importance for model establishment and several criteria should be satisfied: (i) As many as possible compounds should be included to ensure the statistical significance; (ii) The range of biological activity should be wide enough; and (iii) The range of biological activity of the training set and the test set should be same or comparable [30]. The dataset used in this study was collected from the BindingDB database [31], which contains 1174 entries of experimentally measured IC_50_ of FGFR1 inhibition. Finally, a database containing 713 compounds was obtained after filtering redundancy. Then, the dataset was randomly divided into a training set (481 compounds) and a test set (232 compounds) using Discovery Studio 3.0 in a ratio of 2:1. As shown in Figure 7, the biological activity range and median value of training set and test set are similar, suggesting that the dataset we used for QSAR modeling was reasonable.

In view of the structural diversity of FGFR1 inhibitors, it is unpractical to develop a single model to interpret all types of structure-activity relationships. To address the issue, the compounds in training set were firstly divided into different groups according to their core skeletons. If the number of compounds of a group was less than 15, the group was not taken into consideration due to less statistical significance. Finally, eight groups of FGFR1 inhibitors were generated (Table 5). BindingDB IDs of compounds in training set and test set are provided in Supplementary file 1.

**Table 5:** Skeletons and number of molecules in training set and test set of each group.

Group	Skeleton	Size of Training Set	Size of Test Set
1	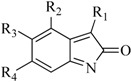	83	54
2	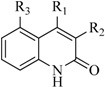	29	16
3	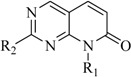	62	29
4	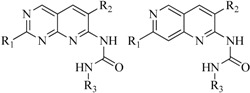	99	43
5	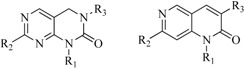	18	9
6	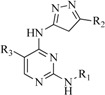	25	16
7	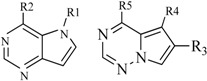	20	23
8	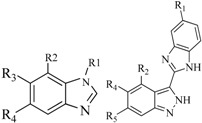	39	42

### 3.2. Pharmacophore Hypothesis Generation

#### 3.2.1. Conformation Analysis

Pharmacophore hypotheses were generated using the Phase 3.1 implemented in the Maestro 9.0 software package (Schrödinger, LLC). Phase translates the ligands into bit strings by applying a tree-based partitioning algorithm and distinguishes multiple binding modes using a bi-directional clustering approach [32]. Since pharmacophore modeling requires all-atom 3D structures to represent the active form of the inhibitor, it is crucial to consider a variety range of conformations so as to increase the possibility of finding the one close to the natural bound structure. Training ligands were firstly cleaned up to ensure the structures are in 3D, and the count ions and water molecules are excluded. Additionally, parameters were set to retain specified chiralities, generate a maximum of 32 stereoisomers and ionize at target pH 7.4. Once the clean-up ligand structures were generated, a conformational search was carried out using the ConfGen module of Maestro with default parameters, to generate a set of conformers for each structure. Potential energy calculation was carried out using the OPLS_2005 force field. An RMSD cutoff of 1.00 Å was used for eliminating redundant conformations.

#### 3.2.2. Generate Common Pharmacophore Hypothesis (CPH)

This procedure contains two steps: (1) Create sites and (2) Find common pharmacophores. In order to develop reliable QSAR models, a training set should contain a widely range of biological activity including high active, moderate active and inactive molecules. The high active molecules were used for pharmacophore modeling, while all of molecules were for QSAR modeling and testing. In this study, pIC_50_ values greater than 7.0 were defined as highly active molecules, pIC_50_ values less than 5.0 were defined as inactive molecules and the remaining in-between values were considered to be moderately active. Pharmacophore sites for ligands including hydrogen bond acceptor (A), hydrogen bond donor (D), hydrophobic group (H), negatively charged group (N), positively charged group (P) and aromatic ring (R) were generated in the step of creating site.

In the step of finding common pharmacophores, pharmacophores of all actives were examined and those containing identical features with similar spatial arrangements were grouped together. In this section, CPHs with 4, 5 and 6 pharmacophore sites were considered. For most of the groups, all the actives must be matched to the CPH. However, if no CPH was found, the number of actives that must be matched would be reset as a number that is less than the total but higher than two-third of the total number of actives. With a tree-based partitioning technique, CPHs were searched according to their inter-site distance.

#### 3.2.3. Score Hypotheses

The function of score hypothesis step measures the quality of alignment and provides a ranking of different hypotheses. The top 10% of the pharmacophore hypotheses were kept according to their survival score, which was combined by alignment score, vector score and volume score with specific weights. Ligands with vector scores above 0.5 Å and RMSD values of inter-site distances to the reference below 1.2 Å were retained. Then, the retained hypotheses were clustered to eliminate those that resemble each other or have very similar scores. One single representative for each cluster was retained for the followed QSAR modeling.

### 3.3. Build QSAR Models

The 3D-QSAR modeling can be classified into atom-based and pharmacophore-based types, in which the structure of ligand is represented by van der Waals models of atoms and pharmacophore features with a specified radius, respectively. The atom-based QSAR model may work well in the case of compounds that contain only limited structural flexibility or have some common structure features, whereas the pharmacophore-based QSAR models may be more appropriate when the structures are highly flexible and exhibit significant diversity [32]. In this study, an atom-based 3D-QSAR model was constructed for each group of compounds as the compounds share the same or similar scaffolds. There are six atom types in atom-based model: hydrogen-bond donor (D), hydrophobic or nonpolar (H), negative ionic (N), positive ionic (P), electron-withdrawing (includes hydrogen-bond acceptors, W), and miscellaneous (X). Each atom of a compound is assigned to a specific atom type and represented by a sphere with the van der Waals radius [19]. Then, cubes with 1 Å grid were defined to cover the space occupied by these aligned conformations, and then assigned to zero or ones based on whether the cube was occupied by atoms or sites. Thus, a molecule can be represented by a string of zeros and ones and treated as a pool of independent variables. Finally, QSAR models were built by adding PLS factors to these independent variables [32]. Here, the PLS factor was set to three to avoid over-fitting. A regression coefficient was assigned to each bit to facilitate the identification of specific chemical features that favorable or unfavorable to the activity. A leave-one-out (LOO) cross validation analysis was performed to evaluate the predictive ability of QSAR model. Finally, a series of 3D-QSAR models were generated.

### 3.4. Construct Combinatorial 3D-QSAR Model

In each group of inhibitors, multiple pharmacophore-based 3D-QSAR model candidates were generated. A filtering procedure was then performed to retain a single QSAR model for each group, based on several statistic parameters, including *R*^2^, *F*, *p* and stability. Finally, the resulted eight pharmacophore-based QSAR models were used in combination when predicting the activity of a new compound. Since there are eight different QSAR models, a test compound can obtain different prediction values. Here, only one appropriate QSAR model was selected for one test compound, which was determined by the chemical similarity between the test compound and the training compounds used to establish the model. Given a test compound, it was compared to each compound in every group of the training set to calculate Tanimoto coefficient (*T*c) of the pairwise chemical similarity. The averaged *T*c values of 8 groups was then sorted, and the corresponding QSAR model of the group yielding the highest average Tc value was selected to predict the activity of the test compound.

### 3.5. Model Validation

The combinatorial QSAR model was firstly validated by an external test set with known activities. To further evaluate our model for virtual screening, namely, the ability of the combinatorial QSAR model in assigning high ranks to the known actives, a decoy dataset composed of 7665 compounds was collected from the SPECS database (http://www.specs.net) in a proportion of 36:1 to the test set, using DecoyFinder [33]. Of note here is that DecoyFinder may introduce potential biases for the ligand-based virtual screening, and it should be compared with other structure-based approaches with caution [34,35]. Compounds in the decoy dataset were also divided into 8 groups based on the similarity comparison described above. Each structure was subjected to ligand preparation using Ligprep and conformations generation using the ConfGen. The corresponding QSAR model was used to predict the activity using the “find matches to hypothesis” option, which finds matches from a database to a selected hypothesis and calculates activity if the hypothesis has a QSAR model. We then calculated enrichment factors (*EF*) of the decoy set to evaluate the ability of identifying actives from inactives. *EF* is calculated by Equation (1) [36]:

(1) EF=HitssNsHitstNt

where *Hit*s_s_ is the number of actives in the selected front fraction of the ranked list, *Hit*s_t_ is the quantities of actives in database, *N*_s_ is the total number of compounds in the selected fraction of the database and *N*_t_ is the quantities of compounds in database.

### 3.6. Pharmacophore-Based Virtual Screening and 3D-QSAR Analysis

In this study, the combinatorial 3D-QSAR model was used to virtually screen the commercial chemical SPECS database. Firstly, the database was filtered by Lipinski rule of 5 to ensure that the selected compounds have better drug-like properties; Secondly, the compounds were prepared using the same procedures in pharmacophore modeling, and each of them was assigned to a specific group for pharmacophore mapping. The mapped compounds were extracted for activity prediction using the combinatorial 3D-QSAR model; Finally, the top ranked 100 compounds with predicted activity more than 5.0 were purchased for experimental evaluation with enzyme assay.

### 3.7. Enzyme Assay

Enzyme-linked immunosorbent assay (ELISA) was used for enzyme assay in this study. Immunoplates were coated with 125 mL/well of 20 mg/mL of enzyme reaction substrate Poly for 12–16 h at 37 °C. Wells were washed with 200 mL of T-PBS and dried. The reaction was initiated by the addition of 50 μL FGFR1 kinase domains recombinant protein in a shaker after ATP buffer (49 μL) and diluted samples (1 μL) being loaded and samples incubated at 37 °C for one hour. The reaction was terminated by adding 100 μL PY99 antibody dilution to each well and incubated in a shaker for 0.5 h at 37 °C. The precipitations were washed three time with T-PBS. Then, 100 μL of HRP-conjugated goat anti-mouse antibodies were added and incubated in the same way. Following three washing cycles in T_PBS, the chromogenic reaction was induced by 100 mL/well of 2 mg/mL OPD developing solution, and the reaction was allowed to proceed for 1–10 min in the dark at 25 °C. The reaction was stopped with 50 μL of 2 M H_2_SO_4_ and absorbance at 490 nm was measured with VERSA max. The IC_50_ values were calculated by fitting with the parameter of the Hill equation.

## 4. Conclusions

FGFR1 is an important therapeutic target for various malignancies and disorders, and it has attracted a lot of attention from pharmaceutical companies and researchers to find novel FGFR1 inhibitors. In this study, we developed an effective strategy for identifying novel FGFR1 inhibitors using pharmacophore-based virtual screening and 3D-QSAR analyses. Various FGFR1 inhibitors were categorized into different groups based on their core structures, and each group was used for pharmacophore and 3D-QSAR modeling. Then, the resulted models were combined to construct a combinatorial 3D-QSAR model, where a new molecule must be assigned to a specific group based on similarity searching prior to the activity prediction using a particular 3D-QSAR model. The performance of the combinatorial 3D-QSAR model has been validated using an external test set and a large decoy dataset. Subsequently, the SPECS database was screened by multiple pharmacophores and 3D-QSAR predictions. Nineteen hits exhibited more than 50% FGFR1 inhibition at 50 μM concentration. Among the nineteen compounds, two compounds appeared over 50% inhibition of FGFR1 and five compounds displayed over 40% FGFR1 inhibition at 10 μM concentration. IC_50_ values of compound **56** and compound **75** were 7.9 and 55.5 μM, respectively. Although the activities are not strong compared to known FGFR1 inhibitors, these compounds possess novel scaffolds not previously reported as FGFR1 inhibitors. Overall, the presented method is a useful alternative to traditional virtual screening methods, and the obtained active compounds provide new chemical starting points for further structural optimization of FGFR1 inhibitors.

## Supplementary Materials

Supplementary materials can be found at http://www.mdpi.com/1422-0067/16/06/13407/s1.
